# Formononetin Attenuates Airway Inﬂammation and Oxidative Stress in Murine Allergic Asthma

**DOI:** 10.3389/fphar.2020.533841

**Published:** 2020-09-04

**Authors:** La Yi, Jie Cui, Wenqian Wang, Weifeng Tang, Fangzhou Teng, Xueyi Zhu, Jingjing Qin, Tulake Wuniqiemu, Jing Sun, Ying Wei, Jingcheng Dong

**Affiliations:** ^1^ Department of Integrative Medicine, Huashan Hospital, Fudan University, Shanghai, China; ^2^ Institutes of Integrative Medicine, Fudan University, Shanghai, China

**Keywords:** asthma, formononetin, airway inflammation, oxidative stress, nuclear factor erythroid 2-related factor 2, nuclear factor kappa β, c-Jun N-terminal kinase

## Abstract

Allergic asthma has been considered as a respiratory disorder with pathological features of airway inflammation and remodeling, which involves oxidative stress. Formononetin (FMT) is a bioactive isoflavone obtained from Chinese herb Radix Astragali, and has been reported to have notable anti-inflammatory and antioxidant effects in several diseases. The purpose of our study was to elaborate the effects of FMT on asthma and the underlying mechanisms. To establish allergic asthma model, BALB/c mice were given ovalbumin (OVA) sensitization and challenge, treated with FMT (10, 20, 40 mg/kg) or dexamethasone (2 mg/kg). The effects of FMT on lung inflammation and oxidative stress were assessed. In OVA-induced asthmatic mice, FMT treatments significantly ameliorated lung function, alleviated lung inflammation including infiltration of inflammatory cells, the elevated levels of interleukin (IL)-4, IL-5, and IL-13, immunoglobulin (Ig) E, C-C motif chemokine ligand 5 (CCL5, also known as RANTES), CCL11 (also called Eotaxin-1), and IL-17A. In addition, FMT treatments eminently blunted goblet cell hyperplasia and collagen deposition, and remarkably reduced oxidative stress as displayed by decreased reactive oxygen species (ROS), and increased superoxide diamutase (SOD) activity. Furthermore, to clarify the potential mechanisms responsible for the effects, we determined the inflammation and oxidation-related signaling pathway including nuclear factor kappa β (NF-κB), c-Jun N-terminal kinase (JNK), and the transcription factor nuclear factor erythroid 2-related factor 2 (Nrf2). FMT treatments appeared to dramatically inhibit the activation of NF-κB and JNK, significantly elevated the expression of heme oxygenase 1 (HO-1) but failed to activate expression of Nrf2. In conclusion, our study suggested that FMT had the therapeutic effects in attenuating airway inflammation and oxidative stress in asthma.

## Introduction

Asthma is a chronic respiratory disorder of the conducting airways where the epithelial barrier working together with the adaptive and innate immune cells respond to a diverse range of exogenous inhaled stimuli such as allergens and air pollutants (Holgate, 2011). Asthma has long been considered as “type-2-high” asthma with hallmark features of airway hyperresponsiveness (AHR), infiltration of inflammatory cells and airway wall remodeling, which contribute to repeated periods of wheezing, chest tightness, and shortness of breath in susceptible people (Lambrecht and Hammad, 2015; Lambrecht et al., 2019). In asthmatic patients, the direct and indirect medical costs bring a heavy burden to their families and society. Inhaled corticosteroids (ICS) and long-acting β2-agonist (LABA) are known as the most common pharmacological options for management of asthma. Although these medications can attenuate airway inflammation and relieve respiratory symptoms, some asthmatic patients respond poorly to corticosteroid-based therapies, and even experience the severe adverse effects (Williams et al., 2004; Aalbers et al., 2016). Consequently, alternative therapeutic options designed to alleviate airway inflammation and remodeling (such as herbal medicines which has long been used for treatment of bronchial asthma) are urgently required and expected to have better efficacy and safety in asthma therapy.

Oxidative stress is involved in the pathogenesis of asthma (Adcock et al., 2008; Dozor, 2010). Allergens exposure are known to increase the production of oxidants like reactive oxygen species (ROS) and reactive nitrogen species (RNS),which contributes to the imbalance between the oxidants and antioxidants, termed as oxidative stress (Kirkham and Rahman, 2006; Mishra et al., 2018). The existing antioxidant system, composed of enzymatic and non-enzymatic molecules, has capability to remove ROS. The antioxidant enzymes include superoxide dismutases (SODs), catalase, glutathione peroxidase (GPx) and glutathione-S-transferase (GST) etc. In addition, heme oxygenase 1 (HO-1) has been considered as a crucial antioxidant protein, and regulated by the transcription factor nuclear factor erythroid 2-related factor 2 (Nrf2) which translocates into the nucleus and initiatives the expression of antioxidant molecules (Uchida et al., 2017; Liu et al., 2019). Furthermore, oxidative stress plays a crucial role in orchestrating airway inflammation and airway remodeling. Findings from previous study have shown that ROS released by mitochondria promoted TGF-β-mediated collagen production (Jaffer et al., 2015). Elevated oxidative stress has been considered as a driving force behind the inflammation and AHR (McGovern et al., 2016). Hence, aside from alleviating airway inflammation and obstruction, suppression of oxidative stress should be considered in asthma therapy.

A growing number of evidences implicate that nuclear factor kappa β (NF-κB) signaling is involved in airway inflammation. In response to allergen challenge, cytokines, chemokines, and bacterial and viral infections, NF-κB acting as a proinflammatory transcription factor binds to the proinflammatory genes promoter region, and thereby upregulates the expression of many mediators and growth factors important in inflammatory cascade of asthma (Adcock et al., 2008; Papi et al., 2013). In addition, enhanced activity of c-Jun N-terminal kinase (JNK) has also been reported in allergen-induced inflammation and remodeling (van der Velden et al., 2014; Wu et al., 2015; Wu et al., 2016).

**Figure 1:**
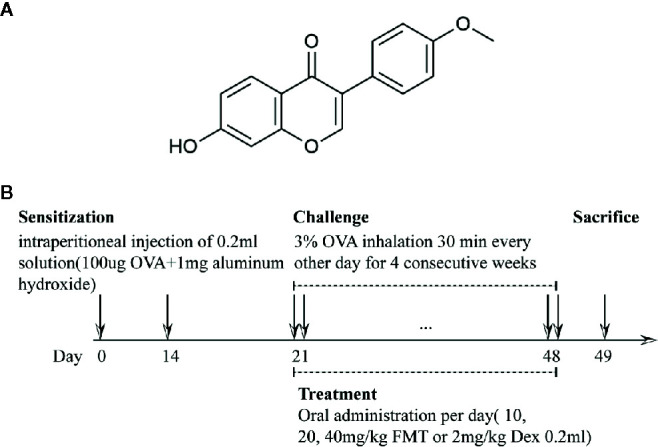
The chemical structure of Formononetin and the flow chart of OVA-induced asthmatic model and treatment **(A)**. The chemical structure of Formononetin (FMT, 7-Hydroxy-4'-methoxy-isoflavone) **(B)**. Asthmatic model and treatment protocol. Mice were grouped, sensitized, challenged, administered and sacrificed from day 0 to 49. Sensitization was performed on day 0 and 14. Challenged and treatment were performed every other day from day 21 to 48. After 24h, mice were sacrificed.

Formononetin (FMT, 7-Hydroxy-4′-methoxyisoflavone) is a bioactive isoflavone isolated from Radix Astragali (the root of *Astragalus membranaceus* var. *mongholicus* or *A. membranaceus*, described as Huangqi in Chinese herb), which has been common applied for treatment of airway inflammation and allergic disease (Shen et al., 2008; Jin et al., 2013; Luo et al., 2014; Luo et al., 2018), whose chemical structure is depicted in **Figure 1**. Our previous studies determined FMT is one of main bioactive ingredients of Bu-Shen-Yi-Qi formula (BSYQF), a Chinese formula widely used to treat respiratory diseases, such as asthma and chronic obstructive pulmonary disease (COPD) (Luo et al., 2014; Nurahmat et al., 2014). FMT has a bioavailability of 21.8% and shows different absorption in all gastrointestinal segments (Luo et al., 2018). An increasing body of evidence suggests that FMT exerts notable anti-inflammatory, anti-allergic, antioxidant, anti-hepatic steatosis, antiproliferative, and anticancer effects *in vitro* and animal models of many disease (Wang et al., 2012; Xu and An, 2017; Fei et al., 2018; Li et al., 2018; Ong et al., 2019; Wang X. S. et al., 2019). Some reports demonstrate that FMT has neuroprotective effect in LPS-stimulated BV2 microglia and high-fat diet-induced cognitive disorder mice (El-Bakoush and Olajide, 2018; Fu et al., 2019; Wang Y. et al., 2019). Recently, FMT has already been reported to exert a significant effect in protecting epithelial integrity *via* G protein-coupled estrogen receptor in an animal model of asthma (Yuan et al., 2020). However, its therapeutic effects to allergic asthma need to be further identified, and their possible mechanisms also need to be further explored. Therefore, our study aims to investigate whether FMT has a therapeutic effect on chronic allergic asthma. To further explore the unrevealed mechanisms, we assess the ability to attenuate lung inflammation and restore the oxidant-antioxidant balance in murine model of allergic asthma.

## Materials and Methods

### Animals

Female SPF BALB/c mice (aged 6 weeks), bought from Shanghai Jiesijie Laboratory Animal Co. Ltd (license number: SCXK (Hu) 2013-0006), were accommodated in pathogen-free cage with constant temperature and humidity, food, and water available. All animal experimental protocols in our study were in accordance with the Guide for the Care and Use of Laboratory Animals, and ratified by the Animal Care and Use Committee of the Fudan University (authorization number: 2018-10-HSYY-DJC-01).

### OVA−Induced Asthmatic Model and Treatment

We established OVA-induced murine model of allergic asthma and set doses of formononetin and dexamethasone based on previous studies with minor modification (Wang et al., 2016; Campa et al., 2018; Li et al., 2018; Luo et al., 2018; Wang X. S. et al., 2019). Sixty mice were randomly assigned to six groups (10 mice/group) as follows: normal control group; asthma group; asthma + formononetin 10 mg/kg group (Winherb Medical Science Co. Ltd, Shanghai, China); asthma + formononetin 20 mg/kg group; asthma + formononetin 40 mg/kg group; asthma + dexamethasone 2 mg/kg group. In the stage of sensitization on days 0 and 14, mice were injected intraperitoneally with 0.2 ml 0.9% saline solution containing 100 µg chicken egg ovalbumin (OVA grade V; Sigma-Aldrich, St. Louis, MO) and 1 mg aluminum hydroxide (Thermo Scientiﬁc). During the period of nasal challenge from day 21, mice were exposed to aerosolized ovalbumin (3% ovalbumin dissolved in 0.9% saline solution) through an ultrasonic nebulizer for 30 min every other day for four consecutive weeks (depicted in **Figure 1**). At the same time, the control group was sensitized and challenged with saline instead of OVA. From day 21 to day 48, formononetin were administered intragastrically with corresponding dose of 10, 20, 40 mg/kg per day. For dexamethasone treatment, mice were given intragastric administration of 2 mg/kg dexamethasone for 28 consecutive days from day 21 to day 48. Apart from that, mice of control and asthma groups were only orally administered with saline.

### Measurement of AHR

24 h after the last OVA challenge, mice were anesthetized with an intraperitoneal injection of 2% phenobarbital sodium (50 mg/kg) to maintain a spontaneous breath. After that, mice were tracheostomized, intubated, and placed in a whole-body plethysmograph connected to the ventilator (Buxco Electronics, Troy, NY) to detect lung function. To evaluate AHR, lung dynamic compliance (Cdyn) and airway resistance (R_L)_ were recorded when mice were exposed to Methacholine (Mch, Sigma-Aldrich; St. Louis, MO) at increasing dose of 0, 3.125, 6.25, and 12.5 mg/ml.

### Assessment of Bronchoalveolar Lavage Fluid

Mice were anesthetized as above, and then intubated with a cannula inside the trachea. Bronchoalveolar lavage ﬂuid (BALF) was collected by intratracheal instillation with 300 µl aliquots of cold PBS twice, centrifuged at 500*g* for 10 min at 4°C. The supernatants were separated and preserved at −80°C for cytokine detection. The cell pellets were resuspended in 50 µl PBS to calculate the number of leukocytes and counts of different populations using an automated cell counter (Hemavet950 instrument; Drew Scientiﬁc Group, UK).

### Lung Histopathology

The left lung was resected, fixed with 4% phosphate-buffered formalin, embedded in paraffin, and cut into 4-µm sections. The tissues were stained with H&E, Masson’s trichrome and Periodic Acid Schiff (PAS). The images were viewed and captured under a light microscopy connecting to a digital camera with a magnification of 100. Inflammation was scored in the light of previous protocols (Kwak et al., 2003). In brief, a subjective value of 0 to 3 was assigned to indicate the degree of inflammation around bronchi or vessels. If no inflammation was detected, a score of 0 was given; if only occasional inflammatory cells were observed, a value of 1 was assigned; if most bronchi or blood vessels were surrounded by a thin layer of (1 to 5 cells thick) inflammatory cells, a score of 2 was set; if most bronchi or blood vessels were surrounded by a thick layer of (more than 5 cells thick) inflammatory cells, a score of 3 was given. Total lung inflammation was expressed as the average of the peribronchial and perivascular inflammation scores.

### Detection of Cytokine, Chemokine, and IgE

IL-4, IL-5, IL-13, IL-17A, eotaxin, CCL-5, IgE levels in BALF were determined by ELISA Kits (Cayman Chemical, Michigan, USA) in accordance with the manufacturer’s protocol.

### Quantification of Lung Th17, Regulatory T Cells, and Eosinophils

Inflammatory cells in lung tissue were analyzed to identify eosinophils (CD45+CD11b+Ly6C-SiglecF+), Th17 cells (CD45+CD4+IL-17A+), regulatory T (Treg) cells (CD45+CD4+CD25+Foxp3+) by flow cytometry as previously described (Zhao et al., 2013; Cossarizza et al., 2017; Dong et al., 2018). Briefly, the excised right lung was digested by using lung dissociation kits (MiltenyiBiotec Technology & Trading Co. Ltd, Shanghai, China) to prepare single cell suspensions following the instructions. And then the cells were stained with the monoclonal anti-murine ﬂuorochrome-conjugated Abs and detected by an Attune NxT instrument (Life Technology). Besides, the antibodies used were bought from BioLegend, including eflour506-labeled anti-CD45, Zombie NIR™ Fixable Viability Kit, Brilliant Violet 421™-labeled anti-CD11b, APC-labeled anti-Siglec-F, PE-labeled anti-Ly-6C, APC-labeled anti-CD25, FITC-labeled anti-CD4, PE-labeled anti-IL-17. PE-labeled anti-Foxp3 was purchased from eBioscience.

### Evaluation of Lung Oxidative Stress

The same weight of the lung tissue homogenates was prepared with 0.05 M Tris–HCl buffer (pH 7.4). The supernatant of homogenates was applied to determine the concentration of SOD and nitric oxide (NO) using commercial kits (Jianchen, Nanjing, China) as manufacturer’s protocol. In addition, frozen lung sections were stained with dihydroethidium (DHE, Sigma-Aldrich; St. Louis, MO) to detect ROS, analyzed by Image-Pro Plus 6.0. At least three 200× fields of image were randomly selected from each lung sections for photographing. The level of ROS was expressed as area density which is the ratio of the integrated optical density value (IOD) to the pixel area of the tissue (AREA).

### Western Blotting

Total protein was extracted from lung tissue. In brief, lung tissues were minced and homogenized in cold RIPA Lysis Buffer containing phosphatase inhibitors and a protease inhibitor, followed by centrifugation at 14,000*g* for 10 min, 4°C. Before adding sample loading buffer, a small part of the supernatant was retained for protein quantitation. The concentration of total protein was detected by Pierce BCA Protein Assay Kit (Thermo Scientiﬁc). Samples were separated by 10% SDS-PAGE and transferred to 0.25 um PVDF membranes. Then, the membranes were blocked with 5% milk at room temperature, incubated with primary antibodies (1:1000) overnight at 4°C and then incubated with HRP-conjugated secondary antibody (1:15000) 1.5 h, and ultimately exposed by LAS-4000 mini (Fujiﬁlm Corporation, Tokyo, Jap). Primary antibodies used in this experiment were purchased from Cell Signaling Technology Inc, including anti-HO-1 Abs, anti-SOD1 Abs, anti-NF-κB Abs, anti-phospho NF-κB Abs, anti-JNK1/2 Abs, and anti-phospho JNK1/2 Abs, except for anti-Nrf2 Abs bought from Proteintech.

### Statistical Analysis

GraphPad Prism 8 (GraphPad Software, La Jolla, CA) was used for all data, which were expressed as the mean ± SD and analyzed by one-way or two-way analysis of variance (ANOVA).When P value < 0.05, it is statistically significant and expressed as: * p < 0.05, ** p < 0.01, and *** p < 0.001.

## Results

### FMT Improves Lung Function in OVA-Induced Murine Asthma

**Figure 2:**
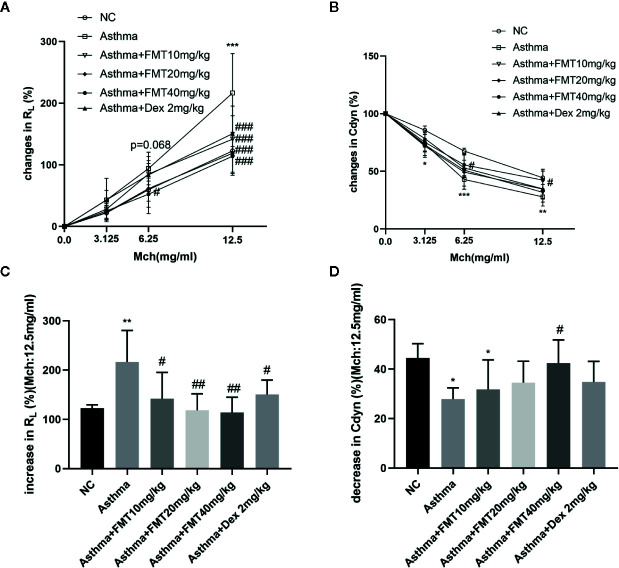
Effect of FMT treatments on lung function in murine asthmatic model. **(A, B)** Increase of lung resistance (RL%) **(A)** and decrease of lung dynamic compliance (Cydn%) **(B)**. Data are shown as the mean ± SD analyzed b two-way ANOVA. **(C, D)** Increase of RL% **(C)** and decrease of Cydn % **(D)** at Mch dose of 12.5 mg/ml. Data are shown as the mean ± SD analyzed by one-way ANOVA. N = 4-6 mice/group. *p < 0.05, **p < 0.01, ***p < 0.001 vs NC group, ^#^p < 0.05, ^##^p < 0.01, ^###^p < 0.001 vs Asthma group.

FMT alleviated AHR in response to the growing doses of Mch as shown in **Figure 2**. In the experiment, asthma group showed a dose-dependent decline in Cydn (**Figure 2B**) and an increase in R_L_ at Mch dose of 6.25 (p = 0.068) and 12.5 mg/ml (p < 0.001) (**Figure 2A**) compared with normal control group, which revealed that mice exposed to OVA have developed significant AHR. Compared with the asthma group, a markedly decreased R_L_ was observed in the mice treated with FMT and Dex at Mch concentration of 12.5 mg/ml (**Figures 2A, C**). The R_L_ of mice in the asthma +FMT 40 mg/kg group also decreased at the Mch level of 6.25 mg/ml (p < 0.05). In addition, FMT exhibited a significant increment in Cydn especially in the asthma +FMT 40 mg/kg group, which was able to gradually normalize Cydn responding to Mch of 6.25 and 12.5 mg/ml (p < 0.05) (**Figures 2B, D**). However, mice in both asthma +FMT 10 mg/kg group and asthma+ Dex 2 mg/kg group also showed a modest increase in Cydn, but these were not statistically significant.

### FMT Attenuates OVA-Induced Airway Inflammation and Remodeling

**Figure 3:**
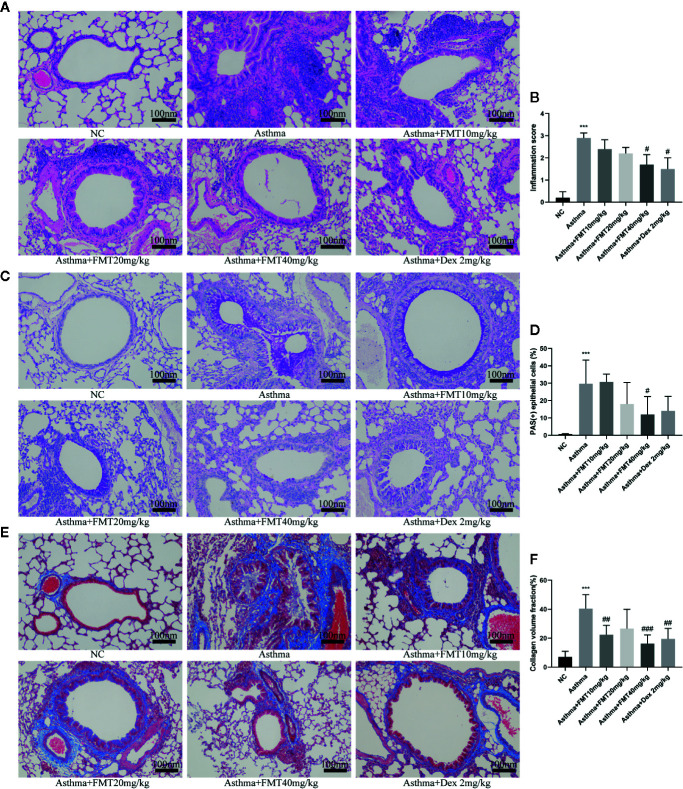
Effects of FMT on OVA-induced airway inflammation and remodeling in lung tissue. (magnification: x200). **(A, B)** H&E-staining and scores of airway inflammation. **(C, D)** Periodic acid-Schiff (PAS) staining and the percentage of PAS + epithelial cells. **(E, F)** Masson’s trichrome staining and collage and volume faction. Bars, 100 μm. Data were shown as mean ± SD analyzed by one-way ANOVA. N = 5 mice/group. ***p < 0.001 vs NC group. ^#^p < 0.05, ^##^p < 0.0.1, ^###^p < 0.001 vs Asthma group.

To identify OVA-induced airway inflammation and remodeling, we detected histological changes in lungs using H&E, PAS, and Masson’s trichrome staining (**Figure 3**). We found that a significant number of inflammatory cells infiltrated into peribronchiolar and perivascular tissues in asthma group compared with NC group (**Figures 3A, B**). In contrast, FMT could remarkably decrease airway inflammation at high dose of 40 mg/kg, similar to asthma+ Dex 2 mg/kg group. In addition, mucus production in the bronchi was evaluated by PAS staining. The results indicated that the lung tissues of asthma group mice showed mucus hypersecretion and a significantly elevated percentage of goblet cells (PAS+ cells) compared with NC group mice (**Figures 3C, D**). However, FMT attenuated mucous secretion and the number of PAS+ cells in asthma +FMT 40 mg/kg group compared with asthma group. The lung tissues of asthma group mice developed a marked increment of collagen deposition in the peribronchiolar areas. In contrast, mice treated with FMT 10 mg/kg or FMT 40 mg/kg or Dex 2 mg/kg showed a significantly decrease in collagen deposition in the peribronchial regions (**Figures 3E, F**).

### FMT Regulates Lung Th17, Treg, and Eosinophils

**Figure 4:**
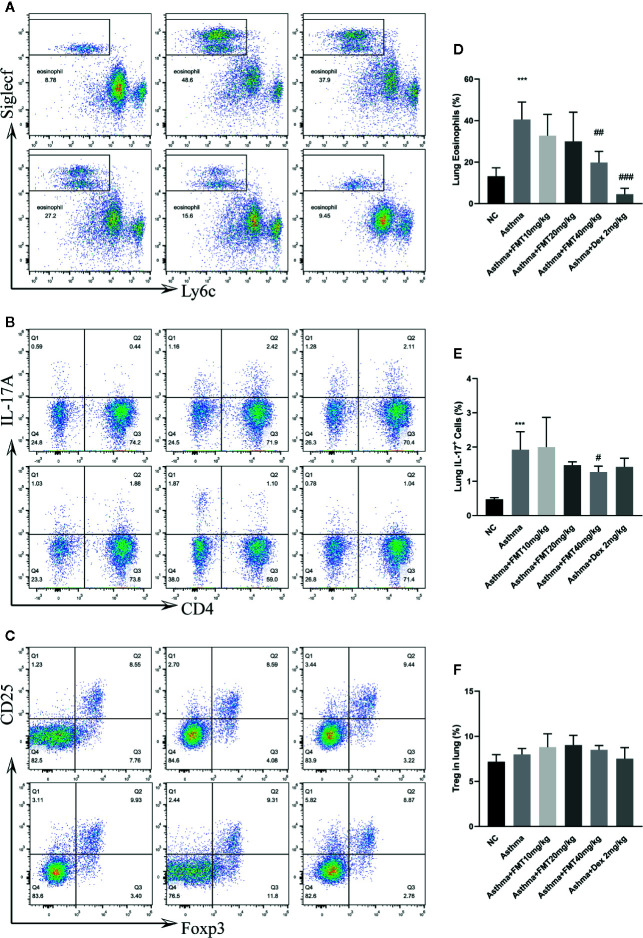
The effect of FMT treatments on lung eosinophils. Th17 and Treg cells in murine asthmatic model. **(A–C)** Flow cytometric identification of lung of each group. **(D–F)** Percentages of eosinophils **(D)**, Th17 cells **(E)** and Tregg cells **(F)** in each group. Bar shows the mean ± SD (samples from 4 to 5 mice/group) ***p < 0.001 vs NC group, ^#^p < 0.05, ^##^p < 0.01, ^###^p < 0.001 vs Asthma group determined use one-way ANOVA.

Previous study revealed that Th2/Th17-predominant asthma was characterized by increased eosinophils but not neutrophils in BALF (Liu et al., 2017). Therefore, we determined Th17, Treg, and eosinophils populations in lung tissue by flow cytometric analysis. We found that the percentage of eosinophils and Th17 cells in asthma group were significantly higher than that in control group (p < 0.05). Administrated with FMT 40 mg/kg or Dex 2 mg/kg, the mice displayed a remarked decrease in the frequency of eosinophils compared with asthma group (p < 0.01 or p < 0.001) (**Figures 4A, B, D, E**). The asthma+ FMT 40 mg/kg group also showed a significantly declining proportion of Th17 cells (p < 0.05) (**Figures 4B, E**). However, no differences were observed in the percentage of Treg cell between any two groups (**Figures 4C, F**).

### FMT Decreases Inflammatory Cells, Ig E, and Cytokines in BALF

**Figure 5:**
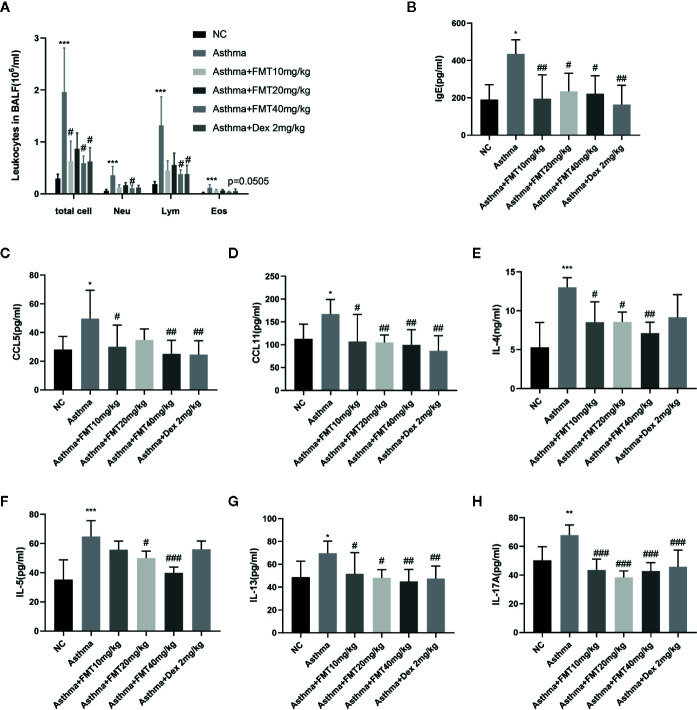
Effects of EMT on inflammatory cells and inflammatory mediators in BALF. **(A)** The number of leukocytes. **(B–H)** The levels of Ig E, CCL5, CCL11, IL-4, IL-5, IL-13, and IL-17A. Data are shown as mean ± SD by one-way ANOVA. N = 4-7/mice/group. *p < 0.05, **p < 0.01, ***p < 0.001 vs NC group, ^#^p < 0.05, ^##^p < 0.001, ^###^p < 0.001 vs Asthma group.

The elevated inflammatory cells infiltration, Ig E level, and cytokines production in BALF are features of allergic airway inflammation. We determined the total number of cells and different populations in BALF. As depicted in **Figure 5A**, asthma group displayed remarkable infiltration of inflammatory cells, such as neutrophils (Neu), lymphocytes (Lym), eosinophils (Eos) (p < 0.0001). FMT 40 mg/kg induced a significant decline in the number of total cell, Neu, Lym (p < 0.05), and a modest decrease in Eos (P = 0.505) compared with asthma group. Besides, Dex significantly reduced the total cell and Lym counts (p < 0.05). FMT 10 mg/kg also diminished the total cell number (p < 0.05).

The above results from flow cytometric analysis of lung tissue and quantification of BALF pointed out that Th2 cells worked together with eosinophils and Th17 cells in the mechanism of OVA-induced asthma. Therefore, we measured some chemokines and mediators related to migration and infiltration of eosinophils, including IgE, CCL5 (also called RANTES), CCL11 (also called Eotaxin). The results demonstrated the significant elevation of total Ig E, CCL5, and CCL11 level in asthma group compared with control group (p < 0.05). However, this increment was abolished by FMT and Dex treatment (**Figures 5B–D**). It is now generally accepted that Th2 cells and Th17 cells significantly contributed to atopic asthma, hence we detected Th2-derived cytokines—IL-4, IL-5, IL-13, and Th17-derived cytokines—IL-17A in BALF. Our data confirmed this. The findings suggested that OVA exposure contributed to the remarkable elevation of IL-4, IL-5, IL-13, and IL-17A level in asthma group mice compared with control group mice. However, FMT treatments significantly decreased OVA-induced increment of IL-4, IL-5, IL-13, and IL-17A. Dex appeared to exert a slight decrease in IL-4 and IL-5 level but not in IL-13 and IL-17A (**Figures 5E–H**).

### FMT Reduces Oxidative Stress in Lung Tissue

**Figure 6:**
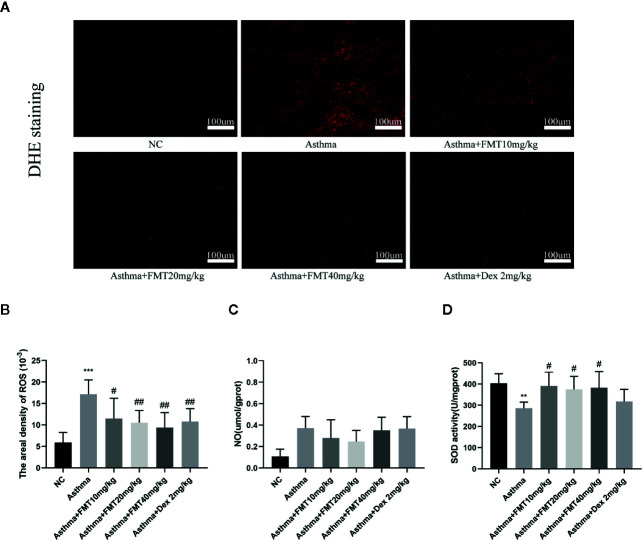
Effects of FMT on oxidative stress in lung tissue. **(A, B)** The level of in situ ROS, stained with DHE in frozen lung tissue (magnification: x200) The fluorescence values are expressed as the areal density, the ratio of the integrated optical density value (IOD) to the pixel area of the tissue (AREA). **(C)** The level of NO. **(D)** Total SOD activity in lung tissue. Data are shown as mean ± SD. N = 4–7mice/group. Bars 100 μm. *p < 0.05, **p < 0.01, ***p < 0.001 vs NC group, ^#^p < 0.05, ^##^p < 0.01 vs Asthma group.

To determine whether oxidative stress is involved in the induction of asthma, and whether FMT alleviates OVA-induced airway inflammation and remodeling through restoring the equilibrium between the oxidants and anti-oxidants, we examined the oxidative stress indicators, including ROS, SOD, and NO. The level of ROS and NO was markedly increased in asthma group mice. However, mice treated with FMT exerted noticeable amelioration in ROS level but no effect in NO production (**Figures 6A–C**). Apart from inhibiting OVA-induced ROS generation, FMT also promoted pulmonary antioxidant defense. FMT treatment significantly restored OVA-induced reduction of SOD in lung tissue where all doses protected lung from oxidative injury (**Figure 6D**).

### FMT Inhibits NF-κB and JNK Signaling Pathway

**Figure 7:**
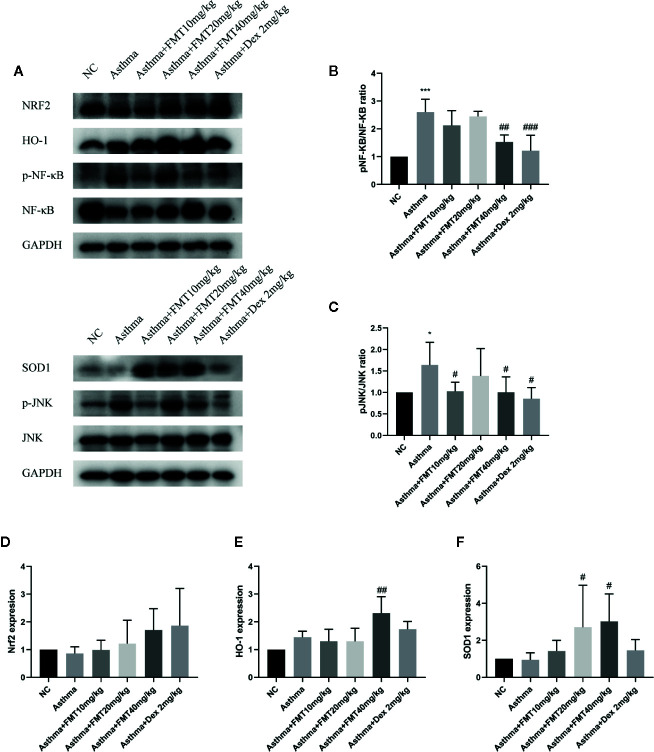
Effect of FMT on inflammation and oxidative stress signaling pathway. **(A)** The protein expression of NF-κB and JNK, as well as Nrf2/HO-1 and SOD 1, was determined by western blotting. **(B, C)** The relative density quantification of NFkb and JNK. The results were expressed as the ratio of phosphorylated proteins relative to total proteins. **(D–F)** The relative density quantifications of Nrf2, HO-1, and SOD1. The results were expressed as the ratio of Nrf2, HO-1, and SOD1 relative to GAPDH, respectively. Data were shown as mean ± SD analyzed by one-way ANOVA. N = 4 mice/group. *p < 0.05, ***p < 0.001 vs NC group, ^#^p < 0.05, ^##^p < 0.01, ^###^p < 0.001 vs Asthma group.

To explore the signaling mechanisms about the effects of FMT on lung inflammation, the phosphorylation statuses of NF-κB and JNK were evaluated (**Figure 7**). The results displayed that NF-κB and JNK phosphorylation were remarkably activated in asthma group mice compared with control group mice. However, the phosphorylation of NF-κB and JNK were significantly suppressed after FMT 40 mg/kg treatment, similar to Dex 2 mg/kg treatment. FMT 10 mg/kg administration also enormously blunted the activation of JNK but failed to affect NF-κB activation. FMT 20 mg/kg appeared to be ineffective at inhibiting phosphorylation of NF-κB and JNK (**Figures 7A–C**).

### FMT Activates Nrf2 Signaling Pathway and SOD1 Expression

To investigated whether FMT mitigates OVA-induced airway inflammation and remodeling by scavenging oxidants, we measured anti-oxidative signaling—Nrf2, HO-1, and SOD1 protein expression in lung tissue (**Figure 7**). The results demonstrated that the expressions of Nrf2 and SOD1 were slightly decreased while the expression of HO-1 was moderately increased in asthma group mice compared with control group, but there were not statistical significance. However, compared with asthma group, the asthma + FMT 40 mg/kg group mice showed a robust increase in the expression of SOD1 and HO-1. Besides, the expression of SOD1 was notably increased in asthma+ FMT 20 mg/kg group mice. Interestingly, the administration of FMT and Dex increased the expression of Nrf2 but that was not statistically significant. Apparently, Dex failed to affect the expression of SOD1 and HO-1 (**Figures 7A–F**).

## Discussion

BSYQF has frequent application in clinical treatment of respiratory diseases such as asthma. Our previous research suggested that BSYQF exerted anti-inflammatory, anti-oxidant and anti-remodeling effects in asthma, and we identified sixteen main bioactive components including FMT (Luo et al., 2014; Nurahmat et al., 2014; Cui et al., 2019). As previously demonstrated, FMT has therapeutic properties in hyperlipidemia and obesity (Fu et al., 2019; Wang Y. et al., 2019), cancers (Hu et al., 2019; Tay et al., 2019) and inflammatory diseases (Li et al., 2018; Aladaileh et al., 2019; Luo et al., 2019; Wang X. S. et al., 2019). Recent reports have demonstrated that FMT alleviated atopic contact dermatitis and allergic asthma by protecting epithelial integrity (Li et al., 2018; Yuan et al., 2020). These inspiring published findings impeled us to explore how FMT could benefit asthma. In this study, we demonstrated that FMT inhibited the pathological progress of allergic asthma in a murine model with typical features of AHR, leukocytes infiltration, multiple cytokines overexpression, goblet cell metaplasia, and collagen deposition. Moreover, our findings indicated that FMT exerted a promising pharmacological effect in alleviating airway inflammation and lung oxidative injury in OVA-induced asthmatic mice, possibly through inhibiting NF-κB and JNK signaling and augmenting Nrf2 signaling.

AHR and lung inflammation are considered as the most prominent features of asthma, constantly evidenced by increased R_L_, decreased lung Cydn and leukocytes infiltration *in vivo* experiments, which has led to the use of bronchodilators and corticosteroids as the mainstream of asthma treatments (Adcock et al., 2008; Lambrecht and Hammad, 2015). A previous study indicated that FMT 10 mg/kg administrated by intraperitoneal injection could significantly alleviate AHR and decrease Th2 cytokines (IL-4, IL-5, and IL-13) levels in the HDM-induced inflammation of allergic asthma (Yuan et al., 2020). Here, we developed an OVA-induced asthma model following by FMT or Dex administration and performed lung function, lung histopathology, and BALF analysis. Our results also confirmed that FMT could suppress AHR, alleviated leukocytes infiltration, mucus hypersecretion, and collagen deposition. However, FMT 10 mg/kg and FMT 20 mg/kg seemingly have modest effects or even have no effect in OVA-induced inflammation and AHR of allergic asthma. The optimal effective dose of FMT appeared to be 40 mg/kg, similar to the effects of Dex 2 mg/kg. That is because FMT was administrated intragastrically not intraperitoneally to mice, potentially consistent with the published data that the pharmacokinetics and bioavailability of formononetin between oral and intravenous administration are different (Luo et al., 2018). Interestingly, BALF cell analysis demonstrated that FMT seemingly exerted a modest decrease in eosinophils counts without statistical significance, potentially a limitation of cell counting in BALF. To identify whether FMT could alleviate eosinophils-rich inflammation in lung, we conducted flow cytometric analysis of lung tissues. We found that FMT reduced lung eosinophils infiltration by nearly half at a dose of 40 mg/kg. The airway recruitment of inflammatory cells is associated with the chemokines. CCL11 (Eotaxin) and CCL5 (RANTES) have been shown to be served as potent chemoattractant for inflammatory cells, especially eosinophils, and is highly expressed in the allergic airway inflammation (Berkman et al., 1996; Chihara et al., 1997; Geslewitz et al., 2018). It has also been reported that the two chemokines have effects on lung fibroblast migration correlated with airway remodeling (Puxeddu et al., 2006; Zhou et al., 2012; Isgro et al., 2013). Indeed, we conﬁrmed the elevated expression of CCL5 and CCL11 in BALF samples from OVA-induced asthma model. FMT was able to remarkably inhibit CCL5 and CCL11 overproduction. These data indicated that FMT exhibits extraordinary anti-inflammatory effects in eosinophils-rich inflammation.

The renowned type-2-high immunity occurs in almost half of asthma patients, manifested as features of eosinophilia, AHR, the elevation of IL-4, IL-5, IL-13, and Ig E in lung and blood (Fahy, 2015; Lambrecht et al., 2019). The biomarkers of type-2-high asthma are believed to develop the pathological progress of asthma. IL-4 promotes the synthesis of Ig E from B cells, and IL-13 induces goblet cell metaplasia associated with mucus hypersecretion, and IL-5 accelerates influx of eosinophils into airways (Grunig et al., 1998; Xia et al., 2018; Busse et al., 2019; Melo et al., 2019). In addition, IL-17 can drive goblet cell metaplasia and mucus hypersecretion in primary human airway epithelia (Pezzulo et al., 2019).Our results suggested that FMT treatments suppressed OVA-induced inflammatory biomarkers expression of IL-4, IL-5, IL-13, IL-17A, and Ig E, and thereby contributed to the reduction of influx of leukocytes and goblet cell metaplasia evidenced by lung histopathology. Besides, Th17 cells have been reported to participate in the pathological process of asthma, especially airway inflammation and remodeling, and abolished Treg cells activity (Zhao et al., 2013). Some studies reported that Th2 and Th17 inflammation are mutually regulated in asthma, and suppression of Th2 cytokines promotes Th17 response, indicating that combination therapies inhibiting both inflammation patterns may exert optimal efficacy in asthma managements (Choy et al., 2015; Liu et al., 2017). Therefore, based upon flow cytometric detection of Th17 cell population from mice lung tissues and Elisa analysis of Th17-inducing cytokines, we found that FMT markedly inhibited OVA-induced Th17 responses. However, our observations showed that Th17 population accounted for a small proportion of CD4+T cells, potentially consistent with the published data about the frequency of cells expressing T-helper-cell-associated cytokines (Tibbitt et al., 2019). Treg cells play a crucial role in maintaining tolerance to allergens, and thereby contributed to controlling inflammation in asthma (Zheng et al., 2019), but sometimes Treg cells could convert to a pathogenic phenotype (Th17-like cells) that exacerbates airway inflammation (Massoud et al., 2016; Noval and Chatila, 2016). Our results suggested OVA exposure induced immune responses in mice, but this involved Th17 cells and not Treg cells, and FMT treatment in mice did not promote Treg cells activation as evidenced by flow cytometric analysis of lung cells. Thus, we inferred that FMT might regulate Th2 and Th17 dysfunctions to reduce the expression of inflammatory mediators, and thereby attenuate airway inflammation in asthma.

In the pathogenesis of asthma, oxidative stress is associated with inflammation and remodeling (Mahut et al., 2004; Kirkham and Rahman, 2006; Dozor, 2010). Growing evidence suggest that both airway inflammation and remodeling trigger increased ROS and NO production in asthma (Thomassen et al., 1999; Mahut et al., 2004; Silveira et al., 2019), and conversely, inhibition of excessive ROS and RNS level alleviates airway inflammation, goblet cells hyperplasia, collagen production, and AHR induced by OVA (Jaffer et al., 2015; Sebag et al., 2017). Interestingly, NO appears to play a dual role in asthma. Some studies indicate that NO exerted beneficial effects on allergen-induced AHR (Schuiling et al., 1998), inflammatory response (Thomassen et al., 1999), and downregulates NF-κB activity (Raychaudhuri et al., 1999). FMT has been reported to prevent methotrexate-induced acute kidney injury through suppressing oxidative injury as evidenced by decreased ROS and NO production (Aladaileh et al., 2019). Here, we detected the oxidative biomarkers in lung tissue. Consistent with the published studies, we found OVA exposure significantly increased ROS and NO level in asthmatic mice lung, however, FMT attenuated oxidative injury through diminishing *in situ* level of ROS rather than NO. In the lung oxidative biology, the endogenous antioxidant system can eradicate excessive oxidants to avoid oxidative stress. The three SOD proteins acts as the important antioxidant to neutralize and remove ROS, and that includes Cu/Zn superoxide dismutase (SOD1), manganese superoxide dismutase (SOD2) and extracellular superoxide dismutase (SOD3) (Kirkham and Rahman, 2006). Meanwhile, the activity and production of total SOD are attenuated when oxidative stress occurs in lung inflammation (Shen et al., 2017; Ye et al., 2017). SOD1 was reported to effectively account for all SOD activity in BALF, as part of lung antioxidant defense (DiSilvestro et al., 1998). Previous published data suggested mice received FMT 10, 20, or 40 mg/kg exhibited a noticeable enhancement of SOD activity (Aladaileh et al., 2019). In accordance, the activity of SOD was decreased in the lung tissue from OVA-challenged mice. FMT treatment signiﬁcantly boosted it and produced a stronger increase than DEX. Furthermore, we detected SOD1 expression in lung tissues through western blotting, and the data also confirmed this. Thus, our findings concluded that FMT treatment remarkably restoring antioxidant defenses in lung tissue where all doses protected lung from oxidative injury.

To gain more insight into the potential mechanisms of the anti-inflammatory and antioxidant effects of FMT on asthma, we detected inflammation and oxidative stress signaling. NF-κB is known to acts as one of major contributors to inflammatory pathways. The phosphorylation of NF-κB p65 and IκB kinase is highly activated in airway inflammation (Papi et al., 2013; Ye et al., 2017). Consistently, our study found that OVA exposure promoted NF-κB activation in mice from asthma group compared with control group. Moreover, NF-κB activation has been associated with mechanisms of ROS-mediated oxidative stress (Morgan and Liu, 2011; Mishra et al., 2018). Here, OVA exposure induced NF-κB activation and ROS overproduction in lung tissue of asthmatic mice, and that may be explained by the crosstalk between ROS and NF-κB. NF-κB promoted IL-1β–induced nitric oxide synthase expression so as to enhance free radical NO formation, and FMT could inhibit the activation of NF-κB signaling (Wang et al., 2012).Here, Interestingly, a previous study pointed out the role of NO in blocking NF-κB activation including IL-1β, TNF-α production (Raychaudhuri et al., 1999). In this study, upregulated NF-κB signaling was suppressed by FMT, whose efficacy was similar to Dex. In addition, JNK signaling is closely related to airway inflammation. TNF-α or TGF-β1 treatment induced increased phosphorylation of JNK in bronchial epithelial cells (Ma et al., 2016). Inhibition of JNK significantly reduced OVA-induced inflammatory cell infiltration, mucus hypersecretion and cytokine production (Wu et al., 2015; Wu et al., 2016). House dust mite exposure caused less lung collagen deposition in JNK1-/- mice than wild mice (van der Velden et al., 2014). Rhinovirus infection induced glucocorticoid resistance can be reversed by inhibitors of JNK and IκB kinase in asthma (Papi et al., 2013). Here, our data showed that OVA induction of JNK activation was completely suppressed by FMT. Nrf2/HO-1 signaling was known to act as an important part of antioxidant system. Nrf2 activation facilitated the expression of HO-1, the fundamental antioxidant enzyme, to regulate oxidative stress in asthma (Liu et al., 2019). The previous study indicated that HO-1 displayed anti-inflammatory effects in OVA-induced neutrophilic airway inflammation, as demonstrated by inhibition of Th17 cell responses (Zhang et al., 2013). OVA exposure downregulated the Nrf2 signaling, evidenced by the diminished Nrf2 and HO-1 expression (Ye et al., 2017). Nrf2 activator could enhance the lung levels of ROS scavengers and decrease the proinflammatory cytokines release when allergen exposure occurred (Uchida et al., 2017). FMT was reported to enhance Nrf2 signaling and consequent expression of HO-1 and SOD (Aladaileh et al., 2019). Consistent with these studies, our results suggested that FMT remarkably elevated the expression of HO-1, but unfortunately, the increment of Nrf2 was not significant. Absolutely, multiple mechanisms have been involved in the anti-inflammatory and antioxidant effects of FMT. For example, the induction of ROS-mediated oxidative stress and inflammation correlate with mitochondrial dysfunction. Mitochondrial-targeted antioxidant therapy abrogates AHR, inflammation, TGF-β-mediated collagen deposition, decreases ROS production, and downregulates NF-κB activity in allergic asthma (Jaffer et al., 2015; Sebag et al., 2017). However, we do not determine whether mitochondrial structural and functional alteration occurs in asthma and whether FMT could reverse mitochondrial dysfunction to alleviate airway inflammation and oxidative stress. That undoubtedly deserves further exploration in future studies.

## Conclusion

Our study demonstrated the potential therapeutic effect of FMT in the asthmatic murine model, which might be mediated through its anti-inflammatory and antioxidant activities. In summary, our data suggested that FMT exerted anti-inflammatory effects as evidenced by (a) alleviating AHR, inflammatory cell infiltration, goblet cell hyperplasia, and collagen deposition in airways; (b) diminishing elevated expression of IL-4, IL-5, IL-13, IL-17A, Ig E, CCL5, CCL11; (c) reducing Th17 cells and eosinophil recruitment in lung tissue; (d) inhibiting the inflammatory signaling mediated through NF-κB and JNK. Our finding also indicated that FMT restored OVA-induced imbalance of the oxidation and antioxidation as demonstrated by (e) decreasing *in situ* ROS production, increasing SOD activity; (f) upregulating the antioxidative signaling pathway of Nrf2/HO-1. Thus, these results provided new information about the protective effects of FMT against asthma.

## Data Availability Statement

The original contributions presented in the study are included in the article further inquiries can be directed to the corresponding authors.

## Ethics Statement

The animal study was reviewed and approved by the Animal Care and Use Committee of the Fudan University.

## Author Contributions

LY was responsible for the original ideas and designed the experiments. JC, WW, WT, FT, XZ, JQ, TW, and JS assisted LY to conduct experiments. LY analyzed the data and wrote the draft manuscript. YW and JD were responsible to supervise manuscript writing and revised it. All authors contributed to the article and approved the submitted version.

## Funding

This work was supported by grants from the National Natural Science Foundation of China (grants 8177140514, 81774074, 81704154, and 81703829), Shanghai science and technology commission (grants 17401930300 and 18401971300), and Young Elite Scientists Sponsorship Program by China Association for Science and Technology (grant 2018QNRC001).

## Conflict of Interest

The authors declare that the research was conducted in the absence of any commercial or financial relationships that could be construed as a potential conflict of interest.
